# Genetic Architecture of Egg Production Traits in Chickens: A Systematic Review

**DOI:** 10.3390/ijms27125255

**Published:** 2026-06-10

**Authors:** Olga Kochetova, Gulnaz Korytina, Yanina Timasheva, Irina Gilyazova, Anna Chumakova, Alexandra Karunas, Elza Khusnutdinova, Oleg Gusev

**Affiliations:** 1 Institute of Biochemistry and Genetics–Subdivision of the Ufa Federal Research Centre of the Russian Academy of Sciences (IBG UFRC RAS), 450054 Ufa, Russia; guly_kory@mail.ru (G.K.); ianina_t@mail.ru (Y.T.); gilyasova_irina@mail.ru (I.G.); ponchikkpop@yandex.ru (A.C.); carunas@list.ru (A.K.); elzakh@mail.ru (E.K.); 2 Life Improvement by Future Technologies (LIFT) Center, 121205 Moscow, Russia; 3 Intractable Disease Research Center, Graduate School of Medicine, Juntendo University, Tokyo 113-8421, Japan

**Keywords:** *Gallus gallus domesticus*, egg production traits, whole-genome association studies, whole-genome sequencing, transcriptomics, clutch traits, age at first egg, egg number

## Abstract

Egg production in *Gallus gallus domesticus* represents a complex, economically critical trait shaped by multiple interrelated phenotypes, including age at first egg, total egg number, egg weight, and clutch characteristics. These traits are governed by polygenic inheritance and modulated by environmental factors, making the dissection of their genetic architecture essential for improving breeding efficiency, particularly under the emerging “long-life layers” production model. This systematic review aimed to integrate current knowledge on the genetic and molecular basis of egg production traits through analysis of genome-wide association studies and related genomic approaches. A structured literature search identified 27 eligible studies, which were evaluated following PRISMA guidelines. Data extraction and meta-analysis were conducted using standardized genome annotations and computational pipelines. The synthesis of available evidence demonstrates moderate to high heritability for key reproductive traits and highlights consistent genomic signals across multiple chromosomes. Importantly, the findings reveal a shift toward a systems-level understanding of egg production, involving conserved biological pathways related to neuroendocrine regulation, folliculogenesis, and energy metabolism. The integration of diverse genomic approaches enables the development of more precise, breed-specific selection strategies. Overall, these advances support a transition from traditional selection toward molecularly informed breeding frameworks, with significant implications for productivity, sustainability, and global food security.

## 1. Introduction

Egg production in *Gallus gallus domesticus* is a key economically important trait in laying hens, determining the efficiency of flock utilization and the profitability of egg production. Global production of chicken eggs exceeds 1.5 trillion annually, providing one of the most important sources of protein for the world’s population. Traditionally, selection for increased egg productivity has been based on phenotypic data and pedigree analysis; however, advances in molecular genetic technologies have opened new opportunities for understanding the genetic architecture of this complex quantitative trait.

Egg productivity comprises multiple interrelated traits, including age at first egg (AFE), total egg number (EN) during the productive period and EN within specific age intervals, egg weight (EW), and clutch traits that characterize the pattern of egg laying in hens. These include, for example, maximal clutch length (MCL), as well as, in modern systems, egg-laying time (ELT) and the duration of the productive period. Each of these traits is controlled by multiple genes and is influenced by environmental factors, making their study particularly complex [1,2,3].

The rhythmicity and duration of egg-laying cycles are of particular importance in modern breeding, as the genetic determination of these traits underlies the full realization of the bird’s reproductive potential throughout the entire laying period. Traditional studies of genetic parameters have demonstrated moderate to high heritability of clutch traits and strong genetic correlations with EN, which has led to the consideration of traits such as maximum clutch size, average clutch size, and MCL as more informative selection indicators than annual EN alone [1].

At present, the relevance of such studies is driven by the transition to the “long-life layers” model, which aims to achieve up to 500 eggs over 100 weeks of a hen’s life. In this context, alongside total EN, the duration of the productive laying period becomes a key trait of interest. Understanding the genetic architecture of key breeding traits, including the identification of polymorphisms in candidate genes, enables the implementation of marker-assisted selection. This provides opportunities for early prediction of laying persistence, shell quality, age at sexual maturity, and other traits, directly affecting economic efficiency and resource conservation in industrial poultry production.

The onset of sexual maturity is a determining factor for the initiation of egg laying; therefore, AFE is a key predictor of both total productivity and overall reproductive potential in hens. Genetic improvement for this trait in the development of modern egg-type lines and crosses is of particular importance: high uniformity of the commercial flock in the timing of entry into the productive period allows synchronization of technological processes, optimization of the duration of hen utilization, and a substantial increase in total egg output.

The duration of egg-laying cycles and the intervals between them directly influence the number of eggs produced by hens during their productive lifespan. Birds with high egg productivity are typically characterized by long laying cycles and short non-laying intervals.

The productive potential of laying hens and its realization under production conditions depend on numerous factors, including genotype, feeding conditions, and housing environment, such as lighting regimes and microclimate.

Over the past 15 years, with advances in high-throughput genotyping technologies, including SNP arrays and whole-genome sequencing, a substantial body of genome-wide association study (GWAS) data has been accumulated across various chicken breeds. These include commercial high-performance lines such as White Leghorn and Rhode Island Red, as well as indigenous breeds from China, Russia, and other countries [1,2,3]. However, a systematic analysis of these data aimed at identifying common patterns, key genes, and genomic regions has not yet been conducted.

The aim of this systematic review is a comprehensive analysis of current research in the genetics and genomics of egg production in chickens, including GWASs on quantitative egg production traits.

This review summarizes the results of recent GWAS, and quantitative genetic analyses conducted in both specialized laying breeds and local dual-purpose populations, incorporating findings from international research, including Russian-language sources. This systematic literature review will be useful for various stakeholders: it will assist in evaluating performance levels and identifying strategies that egg producers may adopt to extend laying cycles, and it will provide researchers with a comprehensive overview of the current state of knowledge while helping to identify gaps in the scientific literature on egg production.

## 2. Methods

This review was conducted in accordance with the guidelines of the International Journal of Molecular Sciences for review articles. The objective was to integrate and critically evaluate current evidence on the genetic and molecular mechanisms underlying quantitative egg production traits in diverse chicken breeds and commercial crosses. The study protocol was reviewed and approved by the local Ethics Committee, which issued a positive decision (Protocol No. 23, 21 April 2026).

### 2.1. Data Sources and Search Strategy

A comprehensive literature search was performed using the NCBI database (https://www.ncbi.nlm.nih.gov, accessed on 3 April 2026) and Google Scholar (https://scholar.google.com, accessed on 3 April 2026). The search strategy incorporated combinations of the following keywords: chicken, *Gallus gallus domesticus*, egg production, EN, polymorphism, SNP, GWAS, AFE, and clutch traits.

Studies were considered eligible for inclusion if they met the following criteria: (i) original research articles published in peer-reviewed journals; (ii) application of genome-wide association studies (GWAS) or whole-genome sequencing methodologies; (iii) investigation of egg production–related traits, including AFE (AFE), ENx–y (EN across specific production intervals and total output), ACST (average clutch size across intervals), MCST (maximum clutch size across intervals), and MCD (maximum consecutive egg-laying days); and (iv) use of *Gallus gallus domesticus* populations of egg-type or dual-purpose breeding direction.

The final search query combined terms as follows: (“chicken” AND “genome-wide association” OR “genome-wide association study”) OR (“chicken” OR “hen” OR “gallus”) AND (“EN” OR “egg production” OR “egg-laying” OR “AFE” OR “clutch traits”) AND “genome-wide association”.

### 2.2. Study Selection (PRISMA) and Quality Assessment

**Figure 1:**
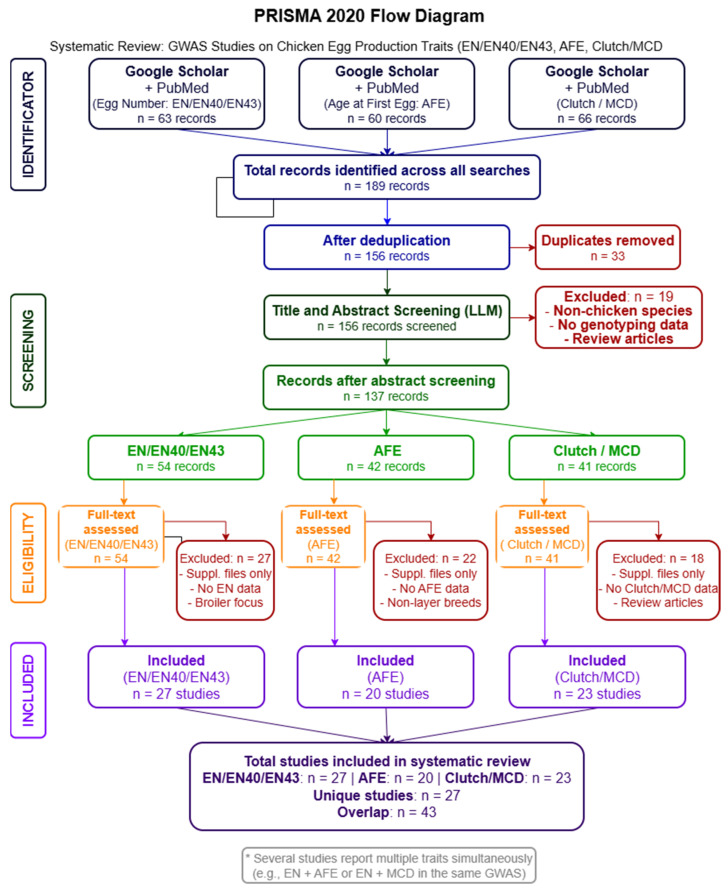
PRISMA 2020 flow diagram. (Image created with diagrams.net (draw.io) software, v. 29.7.8.0, based on author’s data. https://www.diagrams.net/ (accessed on 6 April 2026)).

Study selection was conducted in accordance with the PRISMA 2020 guidelines (https://www.prisma-statement.org, accessed on 25 March 2026). The completed PRISMA 2020 checklist is provided in the Supplementary Materials (Supplementary PRISMA 2020 Checklist). The study selection process was documented using the PRISMA 2020 Flow diagram (Figure 1). During the identification phase, database searches yielded 189 records. After removal of duplicates, 156 records remained for further evaluation.

At the screening stage, titles and abstracts were assessed, leading to the exclusion of 19 records due to irrelevance (including non-genetic studies, studies involving other species, and review articles). Full-text assessment for eligibility resulted in the exclusion of 67 studies, primarily due to the absence of clearly defined phenotypic traits or the use of non-laying chicken populations.

In total, 70 studies met the inclusion criteria and were incorporated into the qualitative synthesis. These studies covered three principal trait categories: EN (EN), AFE (AFE), and clutch-related traits. Of these, 43 studies addressed two or more trait categories, whereas 27 studies focused on a single trait. The study selection process, including reasons for exclusion at the full-text stage, is presented in the PRISMA 2020 flow diagram (Figure 1) and the key studies are summarized in Supplementary Table S1.

### 2.3. Genotyping Methodologies and Study Designs

#### 2.3.1. SNP Array-Based Approaches

The studies included in this review employed a wide range of genotyping platforms and statistical approaches, reflecting the rapid development of genomic tools in poultry research. Early studies (2011–2015) predominantly utilized medium-density SNP arrays, particularly the 60 K Illumina BeadChip (San Diego, CA, USA), comprising approximately 57,636–60,000 markers [4,5,6]. These platforms enabled the first GWAS focused on egg production and related traits. For instance, a GWAS based on the Illumina iSelect 60 K chicken SNP array in White Leghorn and brown-egg dwarf laying hens led to the identification of novel loci associated with egg production and egg quality traits [4]. Subsequent studies further demonstrated the suitability of medium-density arrays for detecting genomic regions influencing reproductive performance [5,6].

In later studies (2015–2026), there was a transition toward the use of high-density SNP arrays (600 K) and whole-genome sequencing, substantially increasing the resolution of genetic analyses. High-density genotyping using the Affymetrix 600 K SNP array (Affymetrix, Santa Clara, CA, USA) in an F2 population derived from reciprocal crosses between White Leghorn and Dongxiang blue-shelled chickens resulted in 435,243 SNPs retained after quality control, enabling the identification of candidate variants associated with egg production traits [7].

A comparable approach was applied using the Affymetrix Axiom HD 600 K SNP array in Chinese commercial Jing Hong laying hens, with additional validation of candidate polymorphisms performed using PCR-RFLP [8]. Further evidence indicates that 600 K SNP arrays provide high-resolution and reliable genotyping, suitable for GWAS, population genetic analyses, and the detection of associations with economically important traits [9].

In addition to high-density platforms, SNP arrays of lower density have also been successfully applied in genomic analyses. For example, the Affymetrix Axiom 50 K SNP array has been used in Rhode Island Red and White Leghorn laying hens for the investigation of clutch-related traits, demonstrating that moderate-density genotyping platforms remain informative for targeted genetic studies [1].

#### 2.3.2. Whole-Genome Sequencing

Whole-genome sequencing (WGS) has emerged as the predominant approach in studies conducted between 2022 and 2026, providing a comprehensive framework for the identification of genetic variants associated with egg production traits. A large-scale study involving 1004 laying hens applied WGS in combination with an additive-dominant GWAS model and integrated ovarian transcriptomic data from 120 individuals [10]. The analysis included four genetic groups—pure-line Beijing-You chickens, White Leghorns, and their reciprocal crosses. By integrating GWAS with ovarian expression quantitative trait loci (eQTL) analysis and transcriptome-wide association studies (TWAS), it was possible to link associated SNPs with gene expression changes, thereby increasing the likelihood of identifying causal variants underlying egg production traits.

This multi-omics strategy led to the identification of 5892 significant SNPs, including 805 additive and 360 dominant variants associated with EN traits [10]. Expression levels of 27 genes were significantly associated with these loci, and four novel candidate genes were highlighted: *TMEM68* (ENSGALG00015011893, mapped to LOC421125 in the NCBI dataset), *DNAH10* (ENSGALG00015026475), *CEP104* (ENSGALG00015025721), and *TGS1* (ENSGALG00015011943), each of which is implicated in fundamental biological processes such as cell structure, division, and reproductive function [10]. The integration of WGS-based GWAS with eQTL and TWAS analyses represents a methodological benchmark for future studies of complex production traits and complements more narrowly focused WGS-based association analyses in specific breeds.

Additional studies have applied WGS to diverse chicken populations with varying levels of egg productivity. For example, WGS-based GWAS in 315 individuals of the indigenous Wuhua breed, characterized by relatively low egg production, identified significant associations across multiple chromosomes [11]. Similarly, WGS was used to investigate egg production traits across different stages of the laying period in Laiwu Black chickens, providing insights into the temporal dynamics of genetic effects [12].

Whole-genome resequencing has also been applied in other populations, including 266 LingKun chickens sequenced using paired-end Illumina technology (PE150) [13], 399 Laiwu Black chickens with the identification of over 10.8 million SNPs [14], 725 Mahuang chickens [15], and 1183 Langya chickens analyzed using low-coverage WGS [16].

WGS provides near-complete coverage of genomic variation, enabling the detection of rare variants as well as structural polymorphisms that are typically inaccessible to SNP array–based approaches, although it remains associated with higher per-sample costs.

#### 2.3.3. Candidate Gene Studies

A number of studies have focused on specific candidate genes identified through prior GWAS findings or based on established biological pathways. For example, the *GARNL1* (*RALGAPA*) gene, which encodes the catalytic alpha subunit of a Ral GTPase-activating protein, has been investigated for its association with reproductive traits in Ningdu Sanhuang chickens [6].

Targeted analyses have also been conducted to validate polymorphisms discovered in genome-wide scans. In particular, SNPs within the *RAPGEF6* (Rap guanine nucleotide exchange factor 6) gene, initially identified through a genome-wide SNP scan, were subsequently examined for their association with egg-laying rate in Chinese Jing Hong laying hens [8].

More recent studies have adopted integrative approaches that combine multiple layers of genomic data. For instance, the integration of GWAS results with eQTL mapping and TWAS has enabled the identification of functional links between genetic variants and gene expression. Using this approach, 27 genes were associated with significant SNPs, and four novel candidate genes were identified [10].

Such integrative strategies enhance the ability to infer causal relationships by connecting genetic variation to gene expression patterns and, ultimately, to phenotypic traits in chickens.

#### 2.3.4. Studied Populations and Experimental Designs

Most studies have focused on purebred lines; however, experimental designs vary considerably, ranging from analyses within individual breeds to F2 intercrosses between genetically divergent populations. For example, an F2 population derived from two divergently selected chicken lines—Russian White and Cornish White—was used to investigate the genetic basis of egg production traits [3]. Such designs maximize genetic variability and enhance mapping resolution, although they may also reveal quantitative trait loci (QTLs) that are specific to the particular cross and not directly transferable to other populations.

In contrast, parallel GWAS conducted in two widely used commercial laying breeds, White Leghorn and Rhode Island Red, enabled a direct comparison of genetic architecture across breeds [9]. This approach provides valuable insight into both shared and breed-specific genetic determinants of egg production traits.

Another study examined seven maternal lines of meat-type chickens from two breeding companies in China, leading to the identification of a significant QTL on chromosome Z with pleiotropic effects on multiple egg production traits [17].

The present systematic review integrates findings from studies published in both English- and Russian-language scientific literature, providing a comprehensive synthesis of current knowledge in the field of reproductive genetics in chickens.

#### 2.3.5. Specialized Laying Hen Breeds

White Leghorn lines represent a highly selected laying hen population characterized by near-maximal peak egg production and extended laying persistence [18]. Consequently, loci associated with EN and laying duration identified in this breed are more likely to reflect fine-scale modulation of neuroendocrine and metabolic regulation within already highly optimized genotypes, rather than large-effect variants segregating in less intensively selected populations. As the dominant commercial laying breed worldwide, White Leghorn chickens feature prominently across the studies included in this systematic review.

One of the earliest GWAS focusing on EN traits in White Leghorn and dwarf laying lines provided initial high-density evidence for loci influencing egg production and quality traits, laying the foundation for subsequent research on laying persistence in commercial populations [4]. Later studies further extended these findings by incorporating whole-genome sequencing approaches, larger sample sizes, and more refined phenotypic definitions of EN traits [2,10,14]. These studies confirmed and expanded the genomic regions initially identified, while reinforcing the importance of specific chromosomal hotspots associated with egg production traits, particularly on GGA2 and GGA5.

White Leghorn chickens were also used as a parental line in an F2 intercross design aimed at dissecting the genetic basis of egg production traits through genome-wide association analysis [7]. In addition, a dedicated GWAS conducted in pure populations of White Leghorn and Rhode Island Red—another major commercial laying breed—enabled direct comparative analysis of the genetic architecture underlying egg-laying performance across distinct genetic backgrounds [9].

Table 1 summarizes the chicken breeds included in this systematic review.

#### 2.3.6. Chinese Indigenous Chicken Breeds

In contrast to highly specialized laying lines, Chinese indigenous chicken breeds are typically dual-purpose populations, selected for both meat and egg production. The majority of studies included in this systematic review focus on such local breeds, including Laiwu Black, Langya, Luhua, Baicheng-You, LingKun, Mahuang, and Wuhua yellow chickens. These populations are generally characterized by moderate egg production, good meat quality, and strong adaptation to local environmental conditions [11,12,13,14,15,16,20]. Compared with intensively selected commercial laying hens, these breeds provide a distinct genetic background that may limit direct transferability of effect sizes but is particularly valuable for identifying loci influencing both EN and laying persistence under less intensive selection pressure.

The Jing Hong chicken is a widely used commercial cross in China, characterized by high egg productivity and brown-shelled eggs [8]. Brown-egg dwarf layers, in turn, represent lines carrying the dwarfing gene (dw), and are notable for reduced feed intake (by approximately 20–30%) while maintaining relatively large egg size, making them economically advantageous [4].

In a targeted GWAS approach, polymorphisms in the *RAPGEF6* gene were validated in Jing Hong chickens using a high-density Affymetrix Axiom HD 600 K SNP array. This gene was associated with egg-laying rate during the late laying period, highlighting its potential role in persistency of production [8].

In contrast, Wuhua yellow chickens, a breed characterized by relatively low egg production, were used to investigate the genetic architecture of key reproductive traits. Traits such as AFE, EN, and clutch size were shown to be controlled by a complex regulatory network involving folliculogenesis and mTOR/insulin signaling pathways. Earlier sexual maturity was additionally associated with larger clutch size and higher EN, indicating coordinated regulation of reproductive timing and productivity [11].

Laiwu Black chickens have also been studied using whole-genome sequencing to investigate egg production traits across different stages of the laying period, providing insight into the temporal genetic architecture of reproductive performance [12].

Additional indigenous populations, such as Gushi chickens, have been investigated using highly integrative multi-omics and statistical frameworks, including SNP-based analyses, linkage analysis, GWAS, canonical correlation analysis-based GWAS, singleton density score methods, multi-tissue transcriptomics, and functional validation along the hypothalamic–pituitary–ovary (HPO) axis. These studies further incorporated hormonal profiling, particularly progesterone (PROG) and estradiol-17β (E2) ratios, as key physiological indicators of reproductive status and egg production efficiency [24].

In LingKun chickens, genome-wide association analyses of egg production and egg quality traits were performed on a population of 226 individuals, covering traits such as body weight at first laying (BWF), EN over 500 days (EN500), egg weight (EW), eggshell thickness (EST), eggshell strength (ESS), and Haugh unit (HU). A total of 37 SNPs showed genome-wide significant associations across these traits [13].

Shuanglian chickens have also been analyzed using whole-genome sequencing for egg production traits, including total EN and maximum consecutive laying days (MCD). These analyses revealed a shared genetic basis between EN40/EN43 and MCD, with a strong QTL on chromosome GGA10 (1.77–1.96 Mb). This region contains a biologically coherent cluster of candidate genes, including *NEO1*, *ADPGK*, *CYP11A1*, and *S1PR4*, which jointly implicate energy metabolism, steroidogenesis, and laying persistence [19].

One of the earlier GWAS on reproductive traits in Chinese indigenous chickens was conducted in Jinghai Yellow chickens using a 60 K SNP array, providing foundational insights into the genetic architecture of reproduction-related traits [22].

More recently, low-depth whole-genome sequencing of a large Langya chicken population (N = 1183) enabled simultaneous analysis of multiple egg production traits, including AFE, stage-specific EN, and maximal laying persistence. This study identified a multifunctional genomic region on GGA5 associated with these traits [16].

In Wenchang chickens, a weighted single-step GWAS (wssGWAS) approach was applied to a large production population, jointly analyzing growth and reproductive traits such as body weight, abdominal fat thickness, total egg production, and AFE. This integrative framework, which combines pedigree, phenotypic, and genomic information, identified 14 candidate genes involved in skeletal development, lipid metabolism, angiogenesis, tissue regeneration, and cellular proliferation and migration. A significant haplotypic QTL associated with body weight was also detected on chromosome Gg4 (75,807,213–75,914,501 bp) [23].

In Mahuang chickens, a combined WGS-based GWAS approach integrating low- and high-coverage sequencing, linkage disequilibrium analysis, KASP genotyping validation, and qRT-PCR expression profiling identified key egg production-associated genes, including *LAMC2*, *RASA3*, and *TRAIP* [15].

In Luhua chickens, genome-wide association analysis of a large population (N = 3151) using a multi-trait animal model identified loci associated with key production traits, including egg weight and number at different laying stages, body weight traits, and AFE. Significant associations were detected with genes such as *ANXA2*, *FZD7*, *CCND1*, and *ADORA2B* [20].

In Baicheng-You chickens, GWAS performed on 742 individuals identified genetic determinants of multiple egg production traits, including first egg weight, body weight at first egg, EN across different laying stages, and maximum consecutive laying days, highlighting substantial genetic heterogeneity within this population [21].

Finally, in an F2 cross between White Leghorn and Dongxiang blue-shelled chickens, egg production traits including EN, laying rate, and AFE were analyzed using high-density 600 K Affymetrix SNP arrays, providing additional insight into the genetic architecture of reproductive performance in divergent crossbred populations [7].

Thus, studies of indigenous chicken breeds provide particularly valuable insights, as these populations represent important genetic resources adapted to local environmental conditions and may harbor unique alleles that are absent in intensively selected commercial populations. In contrast, specialized laying hen breeds have undergone long-term and intensive selection for egg production over many generations, resulting in extremely high productivity but potentially reduced genetic diversity; consequently, their genetic architecture may differ substantially from that observed in less intensively selected populations.

#### 2.3.7. Russian Chicken Breeds and Populations

Within this systematic review, contributions from Russian studies included three investigations of Russian White chickens. One study analyzed an F2 population in which Russian White chickens served as one of the parental lines [3]. Another study conducted one of the first GWAS in this breed, focusing on the yield of extraembryonic fluid and production-related traits. This work applied stringent significance thresholds, validated a key variant (rs13730111) in an independent dataset, and provided evidence for genetic components of adaptation in domestic poultry [5].

In a further study, GWAS was performed to identify genes associated with AFE in laying hens. This analysis focused on a single well-defined reproductive trait, exploited substantial within-population variation, and used an F_2_ resource population, resulting in a clearly defined set of candidate loci on chromosome GGA3 [26].

Collectively, these studies provide an important contribution to poultry genetics, as they focus on breeds adapted to diverse environmental conditions, including low-temperature environments and housing systems that differ substantially from those commonly represented in studies conducted in China and other countries.

#### 2.3.8. Dual-Purpose and Meat-Type Chicken Populations

Dual-purpose and meat-type chicken populations were also included in the systematic analysis to ensure comprehensive coverage of studies addressing the genetic architecture of egg production traits. In this context, analysis of seven maternal lines of meat-type chickens demonstrated that egg production genetics remains relevant even in populations primarily selected for growth traits. A significant quantitative trait locus (QTL) was identified on chromosome Z, influencing egg production traits in meat-type lines, suggesting the presence of partially shared genetic architecture across different production systems [17].

The diversity of chicken populations included in this review reflects both the global importance of egg production and the value of investigating its genetic architecture across a wide range of environmental conditions and selection histories.

## 3. Results and Discussion

### 3.1. Genetic Architecture of AFE

#### 3.1.1. Heritability and Genetic Parameters

Age at first egg (AFE) is a key reproductive trait marking the transition from sexual maturation to the onset of laying. Heritability estimates for AFE generally range from 0.4 to 0.6 in White Leghorn and Rhode Island Red populations, supporting a substantial additive genetic contribution and the feasibility of genetic improvement through conventional and genomic selection [9].

AFE shows strong positive genetic correlations with body weight at first egg and first egg weight, indicating that later-maturing hens tend to have greater body mass at sexual maturity and produce larger initial eggs [9]. In contrast, AFE is negatively correlated with cumulative egg number, consistent with the trade-off between early sexual maturation and long-term egg production.

Together, these findings suggest that AFE is moderately to highly heritable trait influenced by the interplay between reproductive maturation and growth-related processes:

#### 3.1.2. GWAS and Candidate Genes for AFE

GWAS have identified numerous loci associated with AFE across different chicken populations. However, overlap between studies remains limited, suggesting a pronounced breed-specific genetic architecture [9]. For example, no shared candidate genes were detected between White Leghorn and Rhode Island Red populations despite both representing highly selected layer lines [9]. These observations indicate that similar reproductive phenotypes may arise through distinct genetic mechanisms in different populations.

Although the specific candidate genes differ among studies, several recurrent biological themes emerge consistently. Many associated loci involve genes related to neuroendocrine regulation and hypothalamic–pituitary–gonadal (HPG) axis activity. In particular, *NELL2*, *MAPK8*, and *PRLHR* have repeatedly been implicated in reproductive maturation and gonadotropin-releasing hormone-related signalling pathways [2,14,25], supporting a central role for neuroendocrine regulation in determining the timing of sexual maturity.

Additional loci are linked to growth regulation, neuronal signalling, and intracellular signalling pathways. Genes such as *ODZ2 (TENM2)*, *NKAIN2*, *MAP4K3*, and *LIN9* have been associated with AFE in diverse chicken populations, suggesting interactions between growth dynamics, neuronal activity, and reproductive development [4,26].

Several immune-related loci have also been implicated in AFE variation. In particular, TAP2, located within the chicken major histocompatibility complex region on chromo-some GGA16, has been associated with the timing of sexual maturation [7], indicating potential integration of reproductive maturation with broader physiological and immune-related developmental processes.

Overall, current evidence supports a highly polygenic architecture of AFE involving numerous loci with small-to-moderate effects. While overlap at the individual gene level re-mains limited, convergence across studies is considerably stronger at the level of biological pathways, particularly those related to neuroendocrine signalling, growth regulation, and immune function:

A detailed overview of the main candidate genes associated with AFE is presented in Table 2 and Supplementary Table S2.

#### 3.1.3. Biological Interpretation

The limited reproducibility of individual candidate genes across populations under-scores the importance of population-specific genomic analyses in poultry breeding. Mark-er-assisted selection strategies developed in one breed may therefore show reduced predictive accuracy when applied to genetically distinct populations [9].

Despite heterogeneity at the level of individual loci, several conserved biological pathways consistently emerge across studies. Neuroendocrine regulation through the HPG axis, gonadotropin-releasing hormone signalling, MAPK pathways, calcium signal-ling, and immune-related processes appear to represent key regulatory systems underlying reproductive maturation in chickens.

Environmental factors also influence AFE considerably. Photoperiod management and light intensity during rearing affect the timing of sexual maturation, ovarian development, and cumulative egg production [9]. Sensitivity to photoperiodic stimulation differs among breeds, further supporting the importance of genotype-by-environment inter-actions in reproductive regulation.

AFE therefore appears to represent a complex polygenic trait shaped by interactions between endocrine regulation, growth-related processes, metabolic state, and environ-mental conditions.

### 3.2. Genetic Determinants of EN

#### 3.2.1. Heritability Estimates

Egg number (EN) is one of the most economically important traits in layer breeding. Heritability estimates generally range from 0.17 to 0.36 depending on the laying stage and population studied [7]. These moderate estimates indicate that genetic improvement is feasible, although environmental and management factors contribute substantially to phenotypic variation.

Heritability is typically higher during early laying phases, suggesting stronger genetic control during the onset of production than during cumulative lifetime egg output [9]. Available data are consistent with a highly polygenic architecture of EN involving numerous loci with individually small effects [10].

#### 3.2.2. Reproducible Genomic Regions and Biological Pathways

Genome-wide association studies have identified numerous loci associated with egg production traits across chicken populations. However, overlap between studies remains limited, indicating substantial genetic heterogeneity and pronounced breed specificity [9].

Associated loci have been detected on multiple chromosomes, including GGA1, GGA2, GGA3, GGA5, GGA7, GGA16, and chromosome Z. Although the specific causal variants differ between populations, these regions consistently implicate reproductive, metabolic, and neuroendocrine processes.

Despite heterogeneity at the level of individual genes, GWAS findings show stronger convergence at the level of functional enrichment, particularly in signalling pathways, metabolic regulation, and neuroendocrine processes [10].

A summary of genes associated with EN is presented in Table 3 and Supplementary Table S3.

#### 3.2.3. Stage-Specific Genetic Effects

Longitudinal and stage-stratified analyses indicate that the genetic regulation of egg production changes across the laying cycle [7,12,24]. Different genomic regions contribute to early laying onset (21–40 weeks), peak production, and late laying persistence.

A consistent finding across studies is that genetic effects are often stage-dependent rather than stable throughout the production cycle, reflecting temporal changes in endocrine activity, follicular dynamics, and metabolic demands. Longitudinal GWAS further demonstrate that only a subset of loci exert persistent effects across multiple laying phases, whereas many associations are specific to particular stages [12].

#### 3.2.4. Breed-Specific Study Findings

Comparative studies demonstrate limited overlap of candidate loci among White Leghorn, Rhode Island Red, and indigenous Chinese chicken populations [9,15,16,19,20,21,22,23]. These observations indicate that similar production phenotypes may arise through distinct genetic mechanisms.

Population-specific GWAS additionally show that some loci are characteristic of commercial layer lines, whereas others are enriched in indigenous breeds. Only a limited number of loci are consistently replicated across populations, supporting a model in which egg number is influenced by both conserved regulatory pathways and breed-specific variants.

GWAS and sequencing studies indicate that egg production traits are shaped by a highly polygenic, stage-dependent, and population-specific genetic architecture, with most loci exerting only small individual effects and relatively few showing reproducibility across studies or breeds.

#### 3.2.5. Integration with Other Traits

To date, the Chicken QTLdb database compiles information on 29,328 quantitative trait loci (QTL) reported across 420 publications (https://www.animalgenome.org/cgi-bin/QTLdb/GG/index, accessed on 26 March 2026). Analysis of these data reveals a pronounced imbalance in trait coverage: while more than 11,154 QTL have been identified for body weight-related traits, fewer than 3500 are associated with egg production and egg quality parameters. Consequently, the discovery of novel loci and allelic variants influencing productivity at different stages of ontogeny remains a priority. Expanding fundamental knowledge of the genetic architecture underlying egg production is essential for refining genomic selection approaches and enabling a more complete realization of the biological potential of poultry.

A significant QTL affecting egg-laying traits has been identified on chromosome Z across seven maternal broiler lines [17]. The largest QTL detected overall was located on chromosome 4, spanning 78 Mb for body weight, accounting for 32% of genetic variance and exhibiting pleiotropic effects on additional traits. In contrast, the largest effects observed for other traits were considerably smaller, explaining only about 0.9% of the genetic variance. These findings indicate that while certain loci exert broad pleiotropic influences across productivity traits, most QTL associated with egg-laying display relatively modest individual effects. Notably, a relatively compact region on chromosome Z harbors a multilocus QTL influencing egg number across multiple maternal broiler lines.

Within the 10.81–13.05 Mb interval on chromosome Z, 36 genes have been annotated, among which nine are prioritized as candidates for egg production [17]. These include GDNF (glial cell line-derived neurotrophic factor), which supports neuronal survival and function and may contribute to neuroendocrine regulation of reproductive rhythms; DAB2 (DAB adaptor protein 2), involved in endocytosis and signaling pathways of TGF-β and steroid hormones, potentially modulating ovarian and oviductal cellular responses; PRKAA1 (AMP-activated protein kinase catalytic subunit alpha 1), a central sensor of cellular energy status, making it a plausible candidate given the tight link between energy balance and the initiation and maintenance of egg production; NADK2 (mitochondrial NAD kinase 2), which regulates redox processes and may influence energy metabolism in reproductive tissues; WDR70 (WD repeat domain 70), implicated in transcriptional regulation and DNA repair, potentially affecting follicular proliferation and maintenance; LIFR (leukemia inhibitory factor receptor alpha), associated with signaling pathways known to regulate reproductive tissue function; and C6 and C7 (complement components 6 and 7), whose involvement may reflect interactions between immune status and reproductive efficiency. In addition, three single nucleotide polymorphisms (rs318154184, rs13769886, rs313325646) associated with egg number are located within or near the *PRLR* (prolactin receptor) gene, identifying it as another strong functional candidate.

Altogether, this compact region on chromosome Z encompasses genes involved in neurotrophic, hormonal, metabolic, and immune regulatory pathways, along with PRLR, consistent with the complex, multifactorial control of egg production traits.

A genome-wide association study of Russian White chickens examined the yield of extraembryonic fluid (YEF) alongside key production traits in order to assess whether early embryonic characteristics are genetically associated with later egg-laying performance [5]. Significant QTL were identified for YEF and EW, whereas clear associations with overall egg production were not detected, highlighting the complexity of uncovering reliable genetic markers for egg-laying traits in locally adapted populations [5].

### 3.3. Clutch Traits and Maximum Consecutive Days of Laying

#### 3.3.1. Clutch Length and Laying Persistence

The maximum number of consecutive laying days (MCD), clutch length, and related clutch traits reflect the ability of hens to maintain uninterrupted oviposition and are closely associated with laying efficiency. These traits are economically relevant because prolonged clutch sequences reduce the proportion of non-productive days and correlate positively with total egg number [12,27]. In many populations, clutch-related traits show stronger predictive value for long-term productivity than cumulative egg number alone, making them attractive targets for conventional and genomic selection.

Despite their relevance, clutch traits remain less extensively studied than AFE or cumulative egg production. Most genetic analyses have focused on total egg number during defined laying periods, whereas the temporal organisation of laying sequences has received comparatively little attention. Only a small number of genome-wide association studies have specifically investigated clutch length, clutch number, and consecutive non-laying intervals in chickens [14], limiting current understanding of the genetic basis of laying persistence and production stability.

A genome-wide association study in Laiwu Black chickens identified several loci associated with clutch-related traits and MCD, supporting a polygenic basis for laying persistence [14]. Candidate genes included *S1PR4*, *LDB2*, *GRM8*, *NELL2*, *SMYD3*, *SMYD9*, *SPTLC2*, and *PLCL1*, many of which are involved in neuroendocrine signalling, transcriptional regulation, lipid metabolism, and intracellular signalling pathways.

NELL2 and GRM8 participate in neuronal signalling and may influence hypothalamic regulation of reproductive rhythms. PLCL1 is involved in phosphoinositide-mediated signalling associated with hormonal responses, whereas SPTLC2 regulates sphingolipid biosynthesis and membrane-associated signalling processes. Members of the SMYD family, including SMYD3 and SMYD9, function as epigenetic regulators involved in chromatin modification and transcriptional control.

Current evidence supports a model in which clutch traits are shaped by interactions among neuroendocrine, metabolic, immune, and epigenetic pathways. Although still relatively understudied, these traits represent a promising target for improving laying persistence and reducing interruptions in egg production.

#### 3.3.2. Related Traits

Several studies have approached traits associated with the duration of egg laying without directly quantifying the maximum number of consecutive laying days. Instead, they have examined broader aspects of production dynamics that may reflect underlying mechanisms of laying persistence. For instance, a genome-wide analysis investigated egg production efficiency and stability across the laying cycle, focusing on how output fluctuates over time [24]. This study identified *CNNM2*, a gene involved in magnesium homeostasis, as a key candidate. Notably, it was shown to exert a dual effect: increasing variability during the growth phase while stabilizing production during the maintenance phase. Such a pattern suggests the presence of genetic mechanisms that modulate not only productivity but also its temporal consistency, thereby indirectly influencing the duration of sustained egg laying.

In a complementary line of research, a genome-wide association study examined the timing of oviposition in laying hens [28]. These temporal traits are likely linked to circadian regulation of ovulation and may, in turn, affect the continuity of laying sequences. Insights into the daily timing of egg laying therefore contribute to a deeper understanding of the physiological processes that shape clutch length and the number of consecutive laying days.

Large-scale population studies further underscore the complexity of clutch-related traits. An extensive analysis involving Rhode Island Red and White Leghorn populations provided one of the most comprehensive datasets for evaluating clutch parameters at scale [1]. In parallel, QTL mapping on chromosome Z across multiple broiler lines reinforced the importance of sex-linked genomic regions in regulating reproductive traits [17].

Recent work has identified additional candidate genes associated with clutch-related characteristics [11]. Among these, IGF1 has been implicated in clutch size and total egg number through its role in mTOR and insulin signaling pathways. PTK2 (also known as focal adhesion kinase, FAK) has likewise been proposed as a candidate influencing these traits, likely through its involvement in cell adhesion, intracellular signaling, and interactions with metabolic pathways.

In Luhua chickens, genome-wide analysis of the mean number of days within a clutch (MCD) highlighted *SKAP2* and *SAMD4A* as candidate genes [21]. *SKAP2* encodes an adaptor protein associated with Src and integrin signaling, influencing cell adhesion and migration, while *SAMD4A* functions as an RNA-binding translational repressor. Genetic variation in these loci is thought to modulate immunometabolic and post-transcriptional regulatory processes, thereby contributing to the duration and stability of the laying cycle.

A summary of candidate genes associated with clutch traits is presented in Table 4 and Supplementary Table S5.

Clutch traits, encompassing clutch length, clutch number, and the intervals between clutches, provide a more refined characterization of laying patterns than total egg number alone. A GWAS identified 421 significant single nucleotide polymorphisms (SNPs) and 24 candidate genes associated with clutch traits in Laiwu Black chickens [14]. Complementary large-scale analyses demonstrated that these traits exhibit moderate heritability and are genetically distinct from conventional egg production traits in Rhode Island Red and White Leghorn lines [1]. Together, these findings highlight the potential for targeted selection aimed at optimizing not only the quantity but also the temporal pattern of egg production.

### 3.4. Biological Interpretation of Egg Production Architecture

Egg production traits result from the coordinated interaction of multiple physiological systems rather than isolated genetic effects. Although genome-wide studies have identified numerous associated loci, convergence across populations is considerably stronger at the level of biological pathways than individual genes.

#### 3.4.1. Neuroendocrine Control of Reproduction

A central regulatory system underlying egg production is the hypothalamic–pituitary–gonadal (HPG) axis [17]. This network integrates environmental signals, metabolic status, and endocrine cues to regulate reproductive timing, follicular development, ovulation, and laying persistence [7,13,30].

The recurrent identification of genes involved in neuronal signalling and hormone regulation in genome-wide association studies supports the central role of neuroendocrine regulation in reproductive performance. These pathways provide a functional link between central nervous system activity and ovarian physiology, helping coordinate the timing and stability of egg production across the laying cycle.

A summary of the principal biological pathways associated with egg production traits is presented in Table 5 and Supplementary Tables S4 and S6.

#### 3.4.2. Ovarian Development and Follicular Dynamics

Egg production depends on tightly coordinated ovarian follicle recruitment, growth, and maturation. These processes require precise regulation of cell proliferation, differentiation, and DNA replication.

Disruption of follicular development can affect both ovulation timing and overall laying capacity, underscoring the importance of cellular growth regulation in reproductive performance [7,13].

#### 3.4.3. Metabolic and Energy Homeostasis

Sustained egg production is among the most energy-demanding physiological processes in birds and requires continuous coordination of lipid metabolism, mitochondrial function, and systemic energy balance.

Metabolic regulation ensures adequate substrate availability for yolk formation, steroidogenesis, and tissue remodelling, meaning that variation in metabolic efficiency can directly influence laying intensity and persistence [12,29,30]. The available evidence therefore suggests that efficient egg production depends on coordinated regulation of lipid metabolism, cellular energetics, and redox homeostasis.

#### 3.4.4. Calcium Signaling and Oviposition Physiology

Calcium signalling plays a central role in reproductive physiology, particularly in ovulation, eggshell formation, and smooth muscle contraction within the oviduct [31]. Calcium-dependent regulatory systems coordinate endocrine signalling with the mechanical processes required for successful oviposition, linking systemic physiological regulation with reproductive output [30,32,33].

#### 3.4.5. Immune–Reproductive Interaction

Accumulating evidence indicates that immune and inflammatory pathways are closely integrated with reproductive regulation. Immune signalling interacts extensively with endocrine systems, potentially reflecting energetic trade-offs as well as shared regulatory mechanisms linking immunity and reproduction.

These observations support the view that reproductive performance represents a broader systemic physiological phenotype influenced not only by reproductive capacity itself, but also by immune status and inflammatory balance [11,30].

#### 3.4.6. Temporal Plasticity of Genetic Regulation

A defining feature of egg production biology is the temporal plasticity of genetic regulation. The relative contribution of genetic pathways changes across the laying cycle in parallel with shifts in endocrine activity, metabolic demand, ovarian responsiveness, and physiological stress [20,24,25,29].

This dynamic regulation helps explain why different loci predominate during early laying, peak production, and late laying persistence, despite convergence at the level of broader biological functions.

#### 3.4.7. Integrated Model of Egg Production

Egg production can therefore be viewed as a systems-level trait governed by interacting regulatory layers, including central neuroendocrine control through the HPG axis [30,33], ovarian developmental programmes [24,25,29], metabolic and mitochondrial energy supply [24,30], calcium-dependent oviposition mechanisms [31,32,33], and immune–endocrine crosstalk [24,30].

Rather than being determined by a small number of major genes, egg production appears to emerge from distributed regulatory networks whose relative contributions vary across developmental and production stages.

#### 3.4.8. Implications for Genetic Improvement

The pathway-level convergence observed across studies suggests that selection strategies may be more effective when focused on functional regulatory modules rather than individual loci [10,19,24,29,30]. Integration of genomic, transcriptomic, and physiological data will likely be essential for translating genetic findings into predictive breeding models [20,24,25,29,30].

### 3.5. Genomic Regions and Clusters Associated with Egg Production Traits

**Figure 2:**
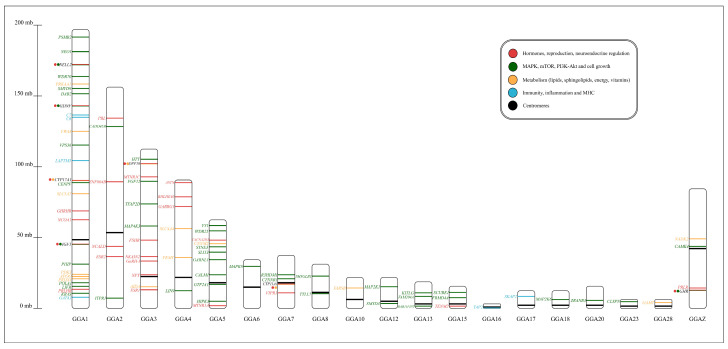
Genomic regions associated with egg production traits. (Image created with diagrams.net (draw.io) software, v. 29.7.8.0, based on author’s data. https://www.diagrams.net/ (accessed on 20 April 2026)).

Multiple genome-wide association and quantitative trait locus studies have identified recurrent genomic regions associated with egg production traits in chickens (Figure 2). Although many loci show population-specific effects, several chromosomal regions have been repeatedly implicated across independent studies and breeds.

Chromosome 1 (GGA1) contains one of the most consistently reported regions associated with egg number, particularly during mid-to-late laying periods. Candidate genes within this region include *POLA1*, *PDK3*, *PRDX4*, and *APOO*, which are involved in cell-cycle regulation, mitochondrial energetics, oxidative stress responses, and lipid metabolism [25]. Additional loci on GGA1 have also been associated with age at first egg [5].

Chromosome 5 (GGA5) represents another major hotspot for reproductive traits. Multiple studies identified loci associated with cumulative egg number, including regions containing *CALM1*, *GARNL1*, *YY1*, *WDR25*, and *SLC25A29* [2,6,7]. These genes are linked to calcium signalling, intracellular transport, mitochondrial metabolism, and transcriptional regulation. Recurrent detection of GGA5 across populations suggests the presence of conserved regulatory regions influencing laying performance.

Additional chromosomes harbour loci with more moderate or population-specific effects. On GGA2, regions involving *GALNT1*, *ZNF704*, and *GHRHR* have been associated with eggshell and reproductive traits [2,4,5]. GGA3 contains candidate loci related to immune regulation and developmental signalling [4]. GGA7 harbours *GRB14*, a regulator of insulin and insulin-like growth factor signalling associated with egg number [4], whereas GGA13 contains the *ODZ2* (*TENM2*) locus linked to sexual maturation [6].

Immune-related regions on GGA16 have also been implicated in reproductive timing. In particular, variants near *TAP2* within the major histocompatibility complex region were associated with age at first egg, supporting interactions between immune and reproductive regulation [7].

The sex chromosome Z also contributes substantially to egg production architecture. Quantitative trait locus analyses in both layer and broiler maternal populations identified regions associated with egg number and clutch-related traits [14,17]. Candidate genes within these regions include *PRLR* and *CAMK4*, which participate in prolactin and calcium-dependent signalling pathways related to ovulation and laying persistence [2,17].

Current evidence indicates that egg production traits are influenced by numerous loci distributed across the genome, with relatively few recurrent regions consistently replicated across studies. These regions predominantly involve pathways related to neuroendocrine regulation, energy metabolism, follicular development, and immune function.

### 3.6. Comparative Population Analysis

#### 3.6.1. Breed-Specific Genetic Architecture

Comparative genomic studies consistently reveal a limited overlap in associated loci and candidate genes across chicken breeds. For instance, genome-wide analyses have shown that candidate genes associated with age AFE do not overlap between White Leghorn and Rhode Island Red populations, despite both being highly specialized laying lines [9]. This pronounced breed specificity suggests that similar phenotypic outcomes may arise through distinct genetic mechanisms, or alternatively, that different allelic variants at separate loci are fixed or segregating across populations.

Such heterogeneity in genetic architecture has important implications for genomic selection. Prediction models trained in one population may exhibit reduced accuracy when applied to another, underscoring the need for population-specific reference panels in genomic prediction frameworks. At the same time, this divergence also creates opportunities for crossbreeding strategies, where complementary genetic architectures may be exploited to enhance performance through heterosis.

#### 3.6.2. Comparison of Layer and Broiler Populations

Comparative analyses between broiler maternal lines and specialized layer lines further highlight both shared and divergent aspects of egg production genetics. In broiler maternal populations, overall egg production levels and underlying genetic architectures differ substantially from those observed in egg-type lines; however, certain key loci, particularly on the Z chromosome, appear to be conserved. The identification of a significant QTL on chromosome Z affecting egg production across seven broiler maternal lines suggests that part of the genetic basis of reproduction is shared across production types [17].

Heritability estimates for egg number in broiler maternal lines are generally low to moderate (0.034–0.258), reflecting historical selection pressure focused primarily on growth and meat yield rather than reproductive performance. In contrast, specialized layer lines typically exhibit higher heritability for egg number and related traits, including clutch length and laying rate. Correspondingly, GWAS in layer populations tend to reveal a more dispersed polygenic architecture, involving genes related to steroidogenesis, follicular development, and neuroendocrine regulation. Notable examples include the TSHR-associated region on chromosome GGA5, the *POLA1–PDK3–PRDX4–APOO* cluster on GGA1, and genes such as *VTG2* and *PDCD11* [12].

These patterns suggest that, in broiler maternal lines, variation in egg production is often concentrated within a limited number of major-effect loci, particularly on the Z chromosome, including regions associated with *PRLR* and related genes. In contrast, layer lines display a more distributed genetic architecture characterized by multiple loci of small to moderate effect across the genome. The relative contribution of specific loci therefore appears to depend strongly on the direction of historical selection pressure.

Understanding both shared and population-specific components of genetic architecture is crucial for the development of breeding strategies, particularly for dual-purpose breeds where a balance between egg production and growth traits is required. Moreover, these comparisons may help reveal fundamental biological constraints and trade-offs underlying reproductive and production traits in chickens.

#### 3.6.3. Geographic Variation and Differences in Management Systems

Studies conducted across diverse geographic regions and production systems, including analyses from Russian research cohorts [3,5,26], provide valuable insight into how genetic architecture may be shaped by environmental adaptation and genotype-by-environment interactions. Populations adapted to distinct climatic conditions, photoperiod regimes, or management practices may therefore exhibit partially divergent genetic determinants of egg production traits.

However, the currently available metadata are insufficient to support robust quantitative comparisons of genetic parameters or locus-specific effects across geographic regions. Future meta-analytical approaches integrating raw genotype and phenotype data from multiple independent studies would be required to systematically address these questions and disentangle environmental from population-specific genetic effects.

## 4. Conclusions

Egg production traits in chickens are shaped by a highly polygenic, temporally dynamic, and breed-specific genetic architecture. Across 27 genome-wide association and sequencing studies, recurrent signals have been identified within a limited number of genomic regions, particularly on chromosomes GGA1, GGA5, GGA7, GGA13, GGA16, and the sex chromosome Z.

Rather than being determined by single major genes, reproductive performance appears to emerge from coordinated interactions among conserved biological systems involved in hypothalamic–pituitary regulation, follicular development, calcium signalling, lipid and energy metabolism, and immune and stress-response pathways.

Clutch-related parameters further extend this framework by capturing the temporal persistence and stability of laying, thereby representing an additional dimension of reproductive efficiency.

Integration of GWAS, transcriptomic, and expression quantitative trait locus data supports a systems-level model of egg production in which regulatory networks and biological pathways are more consistently conserved across populations than individual loci.

The available evidence therefore suggests that reproductive performance in chickens arises from dynamic interactions among neuroendocrine, metabolic, immune, and developmental regulatory systems. Further progress will depend on integrating GWAS, transcriptomics, functional genomics, and longitudinal phenotyping into unified predictive frameworks capable of capturing the temporal and population-specific complexity of egg production biology mandatory.

## Figures and Tables

**Table 1:** Breeds of chickens included in the systematic review.

Breed/Line	Country/Type	Characteristics	Ref.
LingKun	China, indigenous	Large, early-maturing breed with good meat quality, strong disease resistance, and high egg production	[13]
Laiwu Black	China, indigenous, dual-purpose	Slow-growing breed producing light-brown eggs; valued for meat quality and moderate egg production	[14]
Shuanglian	China, indigenous, dual-purpose	Dual-purpose breed noted for adaptability and high genetic diversity	[19]
Wuhua Yellow	China, indigenous	Yellow-feathered local breed with good meat quality, high disease resistance, and relatively low egg production	[11]
Luhua	China, indigenous	Conserved native breed with strong adaptability and stable reproductive performance	[20]
Baicheng-You	China, indigenous	Xinjiang breed characterized by high stress resistance and moderate reproductive performance.	[21]
Jinghai Yellow	China, indigenous	Yellow-feathered dual-purpose breed used as a genetic resource for line improvement	[22]
Langya	China, indigenous	Egg-type breed adapted to local conditions with moderate laying performance	[16]
Wenchang	China, indigenous meat-type	Southern Chinese meat breed with slow growth, highly valued meat quality, and moderate egg production.	[23]
Mahuang	China, indigenous	Yellow-feathered breed adapted to extensive systems, with moderate egg production.	[15]
Ningdu Sanhuang (NDH)	China, indigenous	Three-yellow breed with good disease resistance and moderate egg production; used as a model for reproductive behavior studies.	[6]
Gushi	China, indigenous/improved	Dual-purpose breed with high meat quality, good egg production, and strong adaptability.	[24]
Jing Hong	China, commercial brown-egg layer	High-performing brown-egg layers; used in high-density GWAS of egg production traits.	[8]
Russian White	Russia, specialized egg-type	Developed by crossing local chickens with White Leghorn; characterized by good egg production and cold tolerance.	[3,5]
Cornish White	Russia/international, meat-type	Meat line used as a parental form in F2 crosses with Russian White for genetic studies of egg production.	[3]
White Leghorn	International, commercial egg-type	Highly selected laying breed with peak egg production and long laying cycles.	[4,9,18]
Rhode Island Red	International, commercial egg-type	Large commercial laying breed with high egg production; frequently used alongside White Leghorn in GWAS.	[9,25]
Brown-egg dwarf layers	Commercial dwarf layers	High-producing dwarf lines with reduced feed intake while maintaining large egg size.	[4]
Jing Hong (validation population)	China, commercial cross	Brown-egg high-performance layers used to validate RAPGEF6 effects on egg-laying rate.	[8]
F2 White Leghorn × Dongxiang Blue-shelled	China, F2 resource population	Resource population derived from commercial and indigenous breeds; used for GWAS of egg number (EN), laying rate (LR), and age at first egg (AFE).	[7]

Ref.—reference.

**Table 2:** Candidate genes associated with age at first egg.

Gene(s)	Functional Role	Ref.
*ODZ2*	A neuronal transmembrane surface protein involved in synapse formation, axon guidance, and visual system development; its nuclear fragment can also regulate transcription.	[4]
*GATA3*, *HIPK3*, *RAB11FIP2*, *FAM204A*, *PRLHR*, *NTM*	A set of neuroendocrine and hormonal regulators involved in transcription, signaling, intracellular transport, and cell adhesion, through which the brain and endocrine system may influence sexual maturation and onset of lay.	[25]
*TAP2*	A membrane ABC transporter that, together with TAP1, transports peptides into the endoplasmic reticulum for MHC class I loading and antigen presentation; in chickens, it is located within the MHC region.	[7]
*NELL2*, *FRMD4A*, *PTPN20*, *MAPK8*	A neuronally related signaling group forming an axis linking neuronal signaling, intracellular cascades, changes in cell polarity/behavior, and immune responses, thereby contributing to regulation of AFE.	[13]
*AIDA*, *NKAIN2*, *LIN9*, *MAP4K3*	A cluster of signaling, transcriptional, and ion-regulatory genes on GGA3 affecting growth rate, cell cycle progression, JNK/Wnt signaling, and neuronal activity, which in turn influence sexual maturation and AFE.	[26]
Multiple AFE-associated genes	A set of breed-specific candidate genes reflecting distinct genetic architectures of AFE in White Leghorn and Rhode Island Red, associated with neuroendocrine regulation and reproductive system development.	[9]
*GTF2A1*, *CLSPN*	Regulators of transcription and cell-cycle checkpoints affecting gene expression and replication checkpoint control in ovarian and oviductal cells, indirectly relating to EN and AFE.	[7]

Ref.—reference; AFE—age at first egg; EN—egg number.

**Table 3:** Genes associated with egg number.

Gene(s)	Functional Role	EN Period/Trait	Ref.
*GRB14*	An adaptor protein that binds insulin and IGF receptors and mainly inhibits their signaling; it influences metabolism and ovarian function.	EN	[4]
*GTF2A1*, *CLSPN*	Regulators of transcription and cell-cycle checkpoints that ensure proper gene expression and cell-cycle arrest under replication stress or DNA damage.	EN1	[7]
*FARSB*	The beta subunit of phenylalanyl-tRNA synthetase; it mediates phenylalanine incorporation into proteins and is important for protein synthesis in the egg.	EN2	[7]
*KIAA1549*	A protein known for BRAF fusions and MAPK pathway activation, suggesting a possible role in follicular cell proliferation.	EN3	[7]
*CALM1*	Calmodulin 1, a universal Ca^2+^-binding sensor that regulates numerous calcium-dependent enzymes and channels; it is important for folliculogenesis and ovulation.	EN (21–40 weeks)	[7]
*POLA1*, *PDK3*, *PRDX4*, *APOO*	A group of genes involved in cell-cycle control, energy metabolism, oxidative stress, and lipid metabolism, all of which are critical for ovarian function and sustained egg production.	EN3 (37–50 weeks)	[25]
*GARNL1* (*RALGAPA1*)	A GTPase-activating protein for Ral that regulates small GTPases.	EN300	[6]
*NELL2*, *KITLG*, *GHRHR*, *NCOA1*, *ITPR1*, *GAMT*, *CAMK4*	A neuroendocrine, calcium, and energy-related group of genes that regulate hypothalamic–pituitary signaling, folliculogenesis, and ovarian hormonal responses, as well as calcium signaling and energy supply, thereby affecting the level and dynamics of egg production.	EN	[2]
*IGF1*, *GHR*, *PRLHR*, *NCOA1*	Key neuroendocrine and transcriptional regulators of the GH–IGF1–prolactin/steroid hormone axis; through control of growth, energy metabolism, steroidogenesis, hypothalamic–pituitary activity, and hormone-responsive gene expression in the ovary and other tissues, they collectively shape sexual maturation, follicle development, and sustained high egg production in chickens.	EN	[9]
*ZNF804B*, *DPP10*, *NEO1*, *GABRG1*, *PHIP*, *OSTN*, *GADD45B*, *NFXL1*, *ADAMTS17*	Key genes of the hypothalamic–pituitary–ovarian axis and metabolism, including neuronal receptors and channel proteins, cell-cycle and stress-response regulators, and matrix and growth modifiers.	EN and EN at different stages	[10]

Ref.—reference; EN—egg number.

**Table 4:** Candidate genes associated with clutch traits.

Gene(s)	Functional Role	Clutch Trait(s)	Ref.
*GPCPD1*, *SMYD3*, *SLC4A4*, *FGF12*, *PLD1*, *MAP2K1*, *VWA8*, *MSANTD1*, *HTT*, *PAWR*, *ASRGL1*, *PLCL1*, *CACNA2D3*, *NELL2*, *SMYD9*, *SPTLC2*	A gene set involved in signaling pathways, epigenetic regulation, lipid and amino acid metabolism, and neuronal and calcium signaling; among these, NELL2, SMYD9, SPTLC2, SMYD3, and *PLCL1* were highlighted as the most promising candidates affecting clutch pattern through neuroendocrine, epigenetic, and metabolic regulation.	Clutch traits	[14]
*SMAD9*, *SPTLC2*	A BMP-signaling transducer (SMAD9) and a sphingolipid biosynthesis enzyme (SPTLC2) that may jointly influence clutch intensity through regulation of growth, differentiation, and lipid metabolism.	LR	[14]
*GPC5*, *CKAP2*, *VPS36*, *SH3GLB1*, *MAP2K6*	Genes linked to growth, the cytoskeleton, endosomal trafficking, and stress signaling, influencing body size and condition at the onset of laying and thereby indirectly affecting clutch traits.	BWFE	[14]
*IGF1*, *PTK2*	IGF1 and PTK2 (FAK) are key regulators of growth and mTOR/insulin signaling; their variants are associated with clutch size and egg number, reflecting the influence of growth, metabolism, and cell adhesion on clutch structure.	Clutch size	[11]
*IGF1*, *PTK2*, *SOX5*, *PPFIBP1*, *HIPK2*, *ANKH*, *CAPZA3*, *PLEKHA5*, *NTM*, *MSI2*, *VKORC1L1*	A candidate gene panel affecting growth and proliferation (*IGF1*, *HIPK2*), adhesion and cytoskeleton (*PTK2*, *CAPZA3*, *PLEKHA5*, *PPFIBP1*), mineralization and ion transport (*ANKH*), neuronal regulation of laying rhythm (*NTM*), post-transcriptional regulation (*MSI2*), skeletal development (*SOX5*), and vitamin K-dependent processes (*VKORC1L1*), together providing integrated control of clutch size and structure.	Clutch size	[29]
*SKAP2*, *SAMD4A*	*SKAP2* is a Src/integrin-signaling adaptor involved in cell adhesion and migration, while *SAMD4A* is an RNA-binding translational repressor; variants associated with MCD likely alter immunometabolic and post-transcriptional mechanisms that determine clutch duration and laying persistence.	MCD	[21]

Ref.—reference; LR—laying rate; BWFE—body weight at first egg; MCD—mean clutch duration.

**Table 5:** Key biological pathways associated with egg production.

Biological Pathway	Genes	Traits	Breeds/Lines	Ref. *
Cytoskeleton, cell division, endosomal transport	*CKAP2*, *NCAPG*, *PTK2*, *VPS36*, *SH3GLB1*	BWFE, BM, EW, EN1, EN, ELI	Laiwu Black, Luhua, Baicheng-You, F2 Russian White × Cornish, commercial egg-laying lines	[6,9,14,20,26]
Lipid metabolism; MAPK- and vitamin K-dependent processes	*MAP2K6*, *VKORC1L1*	BWFE, EN43, MCST	Laiwu Black, Luhua, Shuanglian, Wuhua Yellow	[10,14,19,20]
Insulin/IGF and energy metabolism; IP3/Ca^2+^ signaling	*GPC5*, *GRB14*, *PLCL1*, *SLC25A29*, *SLC25A36*	BWFE, EN, AFE, clutch traits, AELT	Laiwu Black, Luhua, White Leghorn, brown-egg dwarf and commercial layers	[4,12,14,20,28]
Neuroendocrine regulation; synapses; GnRH	*NELL2*, *NRSN1*, *NRXN1*, *PPFIBP1*, *MPPED1*	AFE, EN, EN4, EN500	Laiwu Black, Luhua, WL, RIR, LingKun, W2, Wuhua Yellow	[2,3,9,11,14,20]
Transcriptional regulators of growth and body structure	*LCORL*, *LDB2*, *NCAPG*, *CKAP2*	BM, BW, BWFE, WFE, EN1/EN2	RW, Baicheng-You, Wenchang, Shuanglian, Laiwu Black, Luhua	[14,19,20,21,25,28]
Tyrosine phosphatases/kinases and general intracellular signaling	*MAP2K6*, *PTK2*, *PTPN20*	BWFE, EN, ELI, AFE	Laiwu Black, Luhua, Wuhua Yellow, F2 Russian White × Cornish	[3,11,14,20]
Membrane trafficking; autophagy; ESCRT complex	*SH3GLB1*, *VPS36*	BWFE	Laiwu Black, Luhua	[14,20]
Mitochondrial transporters and energy metabolism	*SLC25A29*, *SLC25A36*	EN, AELT	Multi-breed egg-number GWAS, commercial layers	[2,28]
Insulin/IGF adaptors and phosphoinositide signaling	*GRB14*, *PLCL1*	AFE, EN, clutch traits	White Leghorn, brown-egg dwarf/commercial layers, Laiwu Black	[4,12,14]
Neuronal adhesion and transport	*NRSN1*, *NRXN1*, *PPFIBP1*, *MPPED1*	EN500, TEN, EN4, AFE	LingKun, W2, RIR, Wuhua Yellow	[3,9,11,13]

* Ref.—reference; AFE—age at first egg; AELT—age at the end of the laying test; BM—body mass; BW—body weight; BWFE—body weight at first egg; ELI—egg-laying interval; EN—egg number; EN1/EN2/EN4/EN500—egg number measured in the indicated period or dataset-specific interval; EW—egg weight; MCST—mean clutch size trait; WFE—weight at first egg; WL—White Leghorn; RIR—Rhode Island Red; RW—Russian White; W2—Dongxiang Blue-shelled line.

## Data Availability

No new data were created or analyzed in this study. Data sharing is not applicable to this article.

## Supplementary Materials

The following supporting information can be downloaded at https://www.mdpi.com/article/10.3390/ijms27125255/s1.

## Abbreviations

The following abbreviations are used in this manuscript:

AFE	age at first egg
AELT	age at the end of the laying test
BM	body mass
BW	body weight
BWFE	body weight at first egg
ELI	egg-laying interval
EN	egg number
EN1/EN2/EN4/EN500	egg number measured in the indicated period or dataset-specific interval
EW	egg weight
EPT	egg production traits
GWAS	genome-wide association studies
MCD	maximum consecutive egg-laying days
MCL	maximal clutch length
MCST	mean clutch size trait
QTL	quantitative trait locus
RIR	Rhode Island Red
RW	Russian White
SNP	single-nucleotide polymorphism
TWAS	transcriptome-wide association study
W2	Dongxiang Blue-shelled line
WFE	weight at first egg
WGS	whole-genome sequencing
WL	White Leghorn
