# Supplementary material for: Genetic Architecture of Egg Production Traits in Chickens: A Systematic Review
Source: Int J Mol Sci. 2026 Jun 10;27(12):5255. doi: 10.3390/ijms27125255 (PMC13299478; doi:10.3390/ijms27125255)
Supplement: Supplementary file 1 [file ijms-27-05255-s001.zip › Supplementary_PRISMA_2020_checklist.pdf]

PRISMA 2020 Checklist

Section and Topic	Item #	Checklist item	Location where item is reported
**TITLE**			
Title	1	Identify the report as a systematic review.	1
**ABSTRACT**			
Abstract	2	Clear, structured summary: context, objective, methodology, results, conclusion	1
**INTRODUCTION**			
Rationale	3	The rationale described in the context of existing knowledge.	2
Objectives	4	The goals expressed	3
**METHODS**			
Eligibility criteria	5	Inclusion criteria required (i) original research articles published in peer-reviewed journals; (ii) application of genome-wide association srudies (GWAS) or whole-genome sequencing methodologies; (iii) investigation of egg production–related traits, including AFE (AFE), ENx–y (EN across specific production intervals and total output), ACST (average clutch size across intervals), MCST (maximum clutch size across intervals), and MCD (maximum consecutive egg-laying days); and (iv) use of Gallus gallus populations of egg-type or dual-purpose breeding direction.	27
Information sources	6	Articles that included three keywords simultaneously and free full text articles in NCBI were analyzed. Articles in Russian were analyzed in the Google Scholar database.	27
Search strategy	7	The final search query combined terms as follows: ("chicken" AND "genome-wide association" OR "genome-wide association study ") OR (("chicken" OR "hen" OR "gallus") AND ("EN" OR "egg production" OR "egg-laying" OR "AFE" OR "clutch traits") AND "genome-wide association".	27
Selection process	8	The first step was to select publications based on their titles and abstracts. The second step was to select publications by reading the entire articles.	28
Data collection process	9	This review summarizes the results of GWAS studies, and quantitative genetic analysis conducted in both specialized layer breeds and local dual-purpose populations.	27
Data items	10	Recent advances in high-throughput genotyping technologies, including SNP arrays and whole-genome sequencing, have revolutionized our ability to analyze complex quantitative traits. Genome-wide association studies (GWAS) have become the primary approach for identifying genomic regions and candidate genes associated with egg production traits in chickens.	5
Study risk of bias assessment	11	Articles identified through the database search were independently screened by two PhD-level researchers in biological sciences	27
Effect measures	12	p-value based assessments were employed	27
Synthesis methods	13a	According to inclusion criteria and exclusion criteria	27
	13d	Not conducted	
Reporting bias assessment	14	Articles characteristics were presented in the report prior the full text	27

Section and Topic	Item #	Checklist item	Location where item is reported
Certainty assessment	15	recent GWAS articles taking into account all the required information were used.	4-26
**RESULTS**			
Study selection	16	Flow diagram available	Figure S1
Study characteristics	17	Studies characteristics presented	27
Risk of bias in studies	18	Required set of keywords had to be included in the study	1
Results of individual studies	19	To assess the effect and its accuracy, p-values were used.	Table S 6
Results of syntheses	20a	Characteristics of studies presented	27
	20b	The evaluation of genomic and transcriptomic studies addressing egg productivity including age at first egg, total egg number during the productive period and egg number within specific age intervals, and clutch traits that characterize the pattern of egg laying in hens (summarising effect estimates, combining P values).	9-18, Table S 1,2,3,4,6
	20c	Not conducted	
	20d	The study was based on the assessment of the p-value.	Table S 6
Reporting biases	21	Genetic factors for egg productivity including age at first egg, total egg number during the productive period and egg number within specific age intervals, and clutch traits that characterize the pattern of egg laying in hens are presented separately based on the assessment of the p-value.	9-18, Table S 1,2,3,4,6
Certainty of evidence	22	The high level of statistical significance was assessed for each GWAS analysis.	Table S6
DISCUSSION			
Discussion	23a	Provide a general interpretation of the results in the context of other evidence.	4-28
	23b	The study included specialized laying breeds and local dual-purpose populations, incorporating findings from international research, including Russian-language sources.	5-7
	23c	Discuss any limitations of the review processes used.	
	23d	GWAS and whole-genome sequencing (27 studies) reveal a polygenic, age- and breed-dependent genetic architecture of chicken egg production traits: high heritability of AFE and clutch traits, moderate EN, key regions (GGA1,5,7,13,16) and genes (NELL2, GNRH, GRB14/CALM1). Convergence on KEGG pathways (hormonal axes, MAPK/PI3K-mTOR, neuroendocrine regulation) enables genomic selection indices for sustainable productivity.	29
OTHER INFORMATION			
Registration and	24a	Available after ethical committee evaluation of review	

PRISMA 2020 Checklist

Section and Topic	Item #	Checklist item	Location where item is reported
protocol	24b	The review article was evaluated and approved by the local Ethical Committee. A positive conclusion was received, protocol №23 (April 21, 2026).	27
	24c	Describe and explain any amendments to information provided at registration or in the protocol.	
Support	25	The study was supported by the grant from The Ministry of Science and Higher Education of the Russian Federation Agreement No. 075-15-2025-484 dated May 29, 2025.	29
Competing interests	26	No competing interests of review authors.	30
Availability of data, code and other materials	27	Corresponding author	
